# Estimating the Composition of Food Nutrients from Hyperspectral Signals Based on Deep Neural Networks

**DOI:** 10.3390/s19071560

**Published:** 2019-03-31

**Authors:** DaeHan Ahn, Ji-Young Choi, Hee-Chul Kim, Jeong-Seok Cho, Kwang-Deog Moon, Taejoon Park

**Affiliations:** 1 Department of Robotics Engineering, Hanyang University, 55 Hanyang daehak-ro, Ansan 15588, Korea; daehani4@gmail.com; 2 School of Food Science and Biotechnology, Kyungpook National University, 80 daehak-ro, bukgu, Daegu 41566, Korea; chjiyeng91@naver.com (J.-Y.C.); rlagmlcjf124@naver.com (H.-C.K.); kdmoon@knu.ac.kr (K.-D.M.); 3 Food and Bio-industry Research Institute, Kyungpook National University, 80 daehak-ro, bukgu, Daegu 41566, Korea; chojs0988@naver.com

**Keywords:** food analysis, hyperspectral signals, deep neural networks, multimodal learning, autoencoders

## Abstract

There is an increasing demand for acquiring details of food nutrients especially among those who are sensitive to food intakes and weight changes. To meet this need, we propose a new approach based on deep learning that precisely estimates the composition of carbohydrates, proteins, and fats from hyperspectral signals of foods obtained by using low-cost spectrometers. Specifically, we develop a system consisting of multiple deep neural networks for estimating food nutrients followed by detecting and discarding estimation anomalies. Our comprehensive performance evaluation demonstrates that the proposed system can maximize estimation accuracy by automatically identifying wrong estimations. As such, if consolidated with the capability of reinforcement learning, it will likely be positioned as a promising means for personalized healthcare in terms of food safety.

## 1. Introduction

Recently, the World Health Organization (WHO) reported that 38.9% and 8.5% of the global population suffer from obesity and diabetes, respectively. WHO also estimated that 4.2 million deaths were related to these diseases, expecting the numbers to constantly increase [1,2]. Accordingly, smart healthcare for them is rapidly drawing public attention. For instance, those who are sensitive to food intakes and weight changes may want to keep track of the amounts of carbohydrates, proteins, fats, and other nutrients [3]. With smart healthcare, such people will be able to acquire details of food ingredients through their smartphones anywhere, anytime, and anyplace [4,5]. These results may help to monitor and trace what kind of foods affects their health, which is one of the most important information in the personalized and smart healthcare services [6].

It is nontrivial for most people to precisely record the food intake in daily lives, especially when eating unpacked or cooked foods in restaurants as it is difficult to check labels of nutrition facts attached to the packages. Even if accessing the labels, it is a tedious task to manually deal with numbers. This calls for the development of automated techniques to analyze and record food ingredients, preferably supporting the capability of real-time and user-friendly processing.

Conventional ways of analyzing food nutrients were based on chemical methods such as chemical reaction and centrifugation [7,8]. Although these methods produce accurate results, they rely on destructive and time-consuming processes and require pre-configuration in dedicated facilities, which inevitably lead to poor performance in light of flexibility and usability, thereby making it very difficult for users to understand and use the results in daily lives. As another solution, the authors of [9] presented a method based on the radio-frequency identification (RFID) to identify a material when a signal passes through it. However, this method cannot be directly applied to the analysis of food nutrients because the relationship between nutrition factors and RFID features is not yet obvious.

Vision-based methods have been considered as an alternative to counter the drawbacks of conventional methods. They typically rely on pattern matching [10,11,12,13,14] that compares an input image with a reference image to compute their cross-correlation. However, the pattern matching may not perform well on food images because it is vulnerable to changes in environments and viewpoints [15]. By contrast, hyperspectral signals taken in the infrared (IR) range of 900∼2300 nm capture the inherent features irrelevant to the deformation of target, and hence, have been used in chosen topics of food analysis such as the measurement of sugar contents in a fruit [16,17] and the determination of origin country [18,19]. However, it is challenging to apply the pattern matching on hyperspectral signals of food to analyze the food nutrients because most of food hyperspectral signals are relatively similar in shape (i.e., highly correlated with one another), necessitating the development of a new approach.

It is very difficult to precisely define how a hyperspectral signal changes when the composition of food nutrients varies. Nevertheless, the hyperspectral signal is strongly correlated with the amount and ratio of CPF (carbohydrates, proteins, and fats) values, while does not vary with the shape of food or the type of visible light source. This means it is possible to determine the composition of food nutrients in terms of CPF values if there is a sufficiently large number of hyperspectral signals collected from food samples. Motivated by this, we propose a novel system based on deep neural networks (DNN) to estimate CPF values from the hyperspectral signals of food of interest. To the best of our knowledge, this is the first approach to apply DNN to the analysis of food ingredients.

When making a decision, we are usually influenced by previous experiences of same or similar tasks. Inspired by this human nature, our proposed system employs a multimodal architecture tailored to CPF estimation to learn common features from a database of hyperspectral signals. It also incorporates an autoencoder [20] to compress and optimize the common features. In doing so, it can extract key features (e.g., particular peaks around 700∼1100 nm corresponding to the intensity of sugar [21]) by discarding redundant information in raw signals. Moreover, based on the autoencoder, it automatically rejects erroneous CPF estimations due to unseen hyperspectral signals, thereby maximizing the estimation accuracy. This error avoidance capability is very important in maintaining the required level of safety for the system because wrong estimations (e.g., reporting much less calories) may do harm to the users with dietary risks.

For performance evaluation, we obtain hyperspectral signals from 140 real foods in 5 categories and realize 3 DNN-based systems including a full-blown system. Our comprehensive evaluation results indicate that the proposed system generates models that accurately estimate food nutrients as demonstrated by $R^{2}$ values of 0.9543, 0.8527, and 0.8481 for carbohydrates, proteins, and fats, respectively. It also minimizes estimation errors thanks to its error avoidance capability to identify and filter out wrong estimations. Since it is capable of further enhancing the accuracy by learning more hyperspectral measurements, our proposed system will be a promising solution for personalized healthcare from a perspective of food safety.

The remainder of this paper is organized as follows. Section 2 details our proposed system after explaining how to acquire the food hyperspectral signals. Section 3 presents the results of performance evaluation. Finally, the paper concludes with Section 4.

## 2. The Proposed System

### 2.1. Acquisition of Food Hyperspectral Signals

A food hyperspectral signal has a unique-light spectrum in the IR range, in which the wavelength is longer than that of visible light (∼700 nm). We measure and acquire a total of 140 food hyperspectral signals from 5 categories of foods listed in Table 1, in collaboration with the Gangneung Science & Industry Promotion Agency, Korea [22].

To obtain hyperspectral signals, we employ a short-wave IR (SW-IR) apparatus (PANIMA, NIP inc., Gyeonggi-do, Korea) with an IR lens (FA-megapixel, CBC inc., North Carolina, USA). The IR lens can sense a wavelength range of 887∼1722 nm. Using this apparatus, we capture the SW-IR images from multiple regions of a food, and then produce its hyperspectral signal by averaging the values of 5 different regions of food that are randomly selected. The reason for taking an average value is that a pointwise hyperspectral signal is unstable and may vary since the ingredients of the food differ from one region to another.

### 2.2. System Design

In recent years, we have seen exponential growth in the use of DNN that can learn the patterns or features from data by mimicking the mechanism of human brain. The DNN outperformed other machine learning techniques in several applications such as computer vision [23], natural language processing [24], and speech recognition [25]. Hence, we opt to exploit DNN as follows.

**Figure 1:**
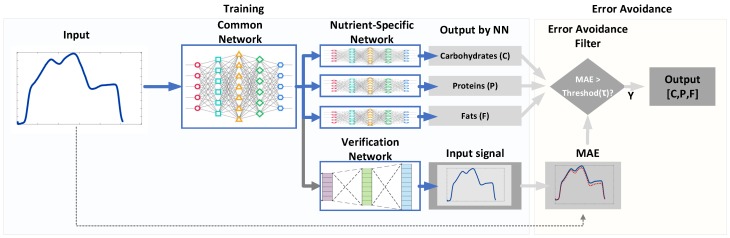
The proposed system for estimating the composition of food nutrients from the measurements of hyperspectral signals.

Figure 1 shows the proposed system that computes the CPF values from a hyperspectral signal of the food of interest. It has three kinds of DNNs that consist of a common network (CN), three nutrient-specific networks (NSNs), and a verification network (VN). The CN accepts an input signal and produces the common features for both NSNs and VN. Based on these features, three NSNs produce each of the CPF estimations, while VN approximates the input signal to verify if estimation results are trustworthy.

### 2.3. Learning Architecture

**Figure 2:**
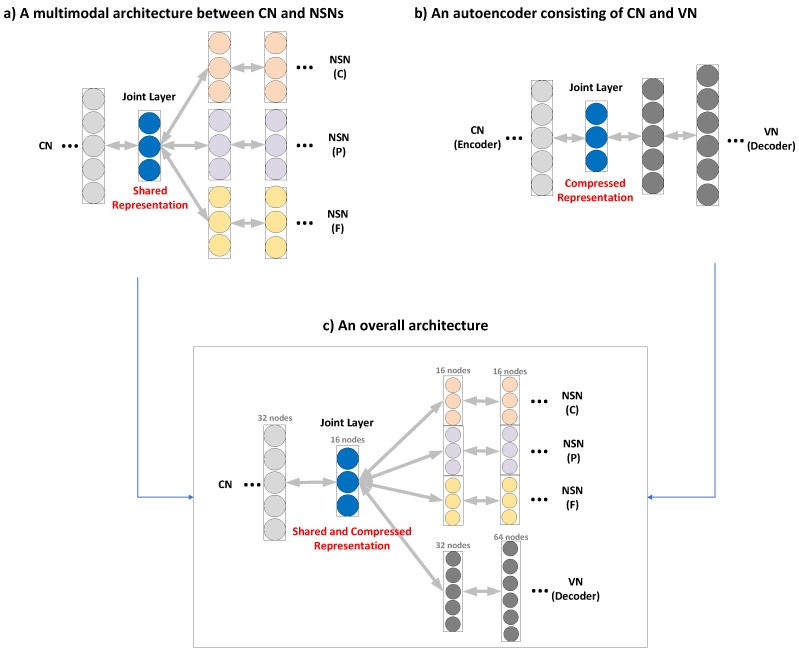
Building blocks of the proposed system: (**a**) a multimodal architecture between CN (common network) and NSNs (nutrient-specific networks), (**b**) an autoencoder consisting of CN and VN (verification network), and (**c**) an overall architecture.

Inspired by the concept of multi-modality [26], we employ a multimodal learning architecture, in which CN and NSNs share a joint layer (JL) in addition to keeping their own hidden layers as shown in Figure 2a. The JL enables the CN to accumulate common features to be shared with NSNs. This means it constructs a shared representation of features via close coupling between the two kinds of networks, and this design choice significantly improves the performance of estimating CPF values.

However, this architecture has a problem, i.e., there might exist redundant information that is less useful in determining the CPF values. It is thus important to make JL as compact as possible. To achieve this, we employ the autoencoder mechanism [20] to learn a *compressed representation of shared features* from a collection of hyperspectral signals. The autoencoder is comprised of an encoder and a decoder as illustrated in Figure 2b. The former takes an input and computes a compressed representation in the JL, doing away with noises and unnecessary information, while the latter reconstructs the input from the compressed representation. The compression of features is implemented by having deeper layers to be smaller than their previous layers. The compressed representation not only reduces the dimensionality but also finds the latent space that best explains the input data or the food hyperspectral signals. Figure 2c presents the overall architecture for multimodal learning. The CN plays the role of an encoder and it produces the compressed representation through a series of hidden layers constructed by applying the sandwich strategy [27], in which the number of nodes of a layer is set to a half of the previous layer. The JL then conveys the thus-constructed compressed representation to the subsequent networks, while the NSNs and VN carry out tasks of estimation and verification, respectively.

To do away with overfitting [28] as well as maximize accuracy, we need to judiciously choose the number of nodes in the JL and NSNs. It is recommended to have at least 17 nodes per layer to ensure reliability [29]. Following this recommendation, we keep reducing the dimension of layers in CN (by a half) until the size of JL reaches 16, which is a multiple of 2 closes to 17. We also set the number of nodes in all layers of NSNs to 16. By contrast, there is no restriction on the number of layers, as will be demonstrated in Section 3.

### 2.4. Error Avoidance

In designing a system for food safety, it is crucial to get rid of inaccurate predictions because such outliers, even if small, could be harmful to the patients or people using the system. While the system in Section 2.3 fits well with the training data of hyperspectral signals, its accuracy may be degraded for unseen signals that deviate significantly from the training data. Training the system with more data may lower the possibility of errors, but it cannot completely avoid the problem. Hence, it is required to automatically detect and reject erroneous results.

**Figure 3:**
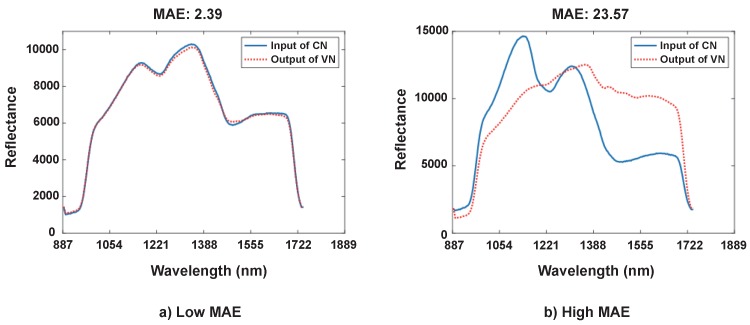
Typical pairs of the input of CN and the output of VN when their mean absolute error (MAE) is: (**a**) small, and (**b**) large.

Motivated by this, we propose an error avoidance scheme based on VN as shown in Figure 1. Thanks to the nature of autoencoder, an output of VN must be similar to its corresponding input of CN if the input signal conforms to the trained system. Otherwise, the output of VN differs from the input of CN. Accordingly, the proposed system benefits from this unique characteristic to detect and remove the outlier, for which it cannot precisely analyze the food nutrients. We use a metric, a mean absolute error (MAE) to quantify the difference of two signals. Figure 3 plots typical pairs of the input of CN and the output of VN. In Figure 3a, the NSNs have a small MAE of 2.39 when the two signals are similar to each other. On the other hand, the MAE increases when they differ from each other as plotted in Figure 3b with an MAE of 23.57. This clearly demonstrates that we can declare an occurrence of error if the two signals do not match well.

We introduce a threshold τ for determining whether to accept the result or not. When we capture multiple hyperspectral signals from the same food sample, the signal slightly differs from others even though producing the same (or highly similar) nutrient values. As such, we need to determine if the difference between hyperspectral signals is acceptable by using Bland-Altman’s study [30]; it provides a range (*mean* ± 1.96 × *standard_deviation*), within which they are statistically considered as the same signal. To specify the range in terms of τ, we analyze 100 foods as follows. For each food, we capture five hyperspectral signals and calculate the average of these signals. We then compute an average MAE between the collected signals and the averaged signal. As a result, we set τ to 9.04 and detect an error if the maximum difference between the two signals is larger than τ. For instance, the input signal in Figure 3a is accepted as its MAE is smaller than τ, while Figure 3b produces an MAE much larger than τ leading to a rejection of the hyperspectral measurement.

## 3. Performance Evaluation

To evaluate the performance, we conducted experiments with 140 real foods listed in Table 1. We randomly chose these foods from the products we usually see at the market. The number of foods meets the requirements of statistical analysis based on Gaussian approximation, i.e., >30 [31] We obtained hyperspectral signals for all these foods by using the apparatus explained in Section 2.1. We used the labels of nutrition facts attached to the food packages as the ground truth values, which means the system was trained for the relationship between measured signals and their associated ground truth values. We did our best to overcome the overfitting problem in the training phase by performing data augmentation that creates several similar data from each of the hyperspectral signals by adding random noises of small magnitude.

For performance comparison we realized 3 systems: (1) a simple DNN-based system, (2) a system based on multimodal DNN, and (3) our proposed system shown in Figure 1. Please note that the first system (DNN) was built by training 3 separate DNNs for each of CPF estimation, while the second system (multimodal DNN) consisted of CN and 3 NSNs sharing JL but with no error avoidance scheme. To quantify the amount of error incurred by these systems, we used a metric, a symmetric mean absolute percentage errors (SMAPE) that converts MAE into a percentage unit; as SMAPE gets closer to 0, the error becomes smaller.

It is crucial to develop a system that fits well with signals that were not taken into account in the training phase. To evaluate this capability, we adopted 10-fold crossvalidation [32] as follows. We divided the dataset into 10 roughly equal subsets, and used 9 of them for training while the rest for testing in the first round of validation. After that, we rotated the roles of validation and test subsets in the next round of validation.

**Figure 4:**
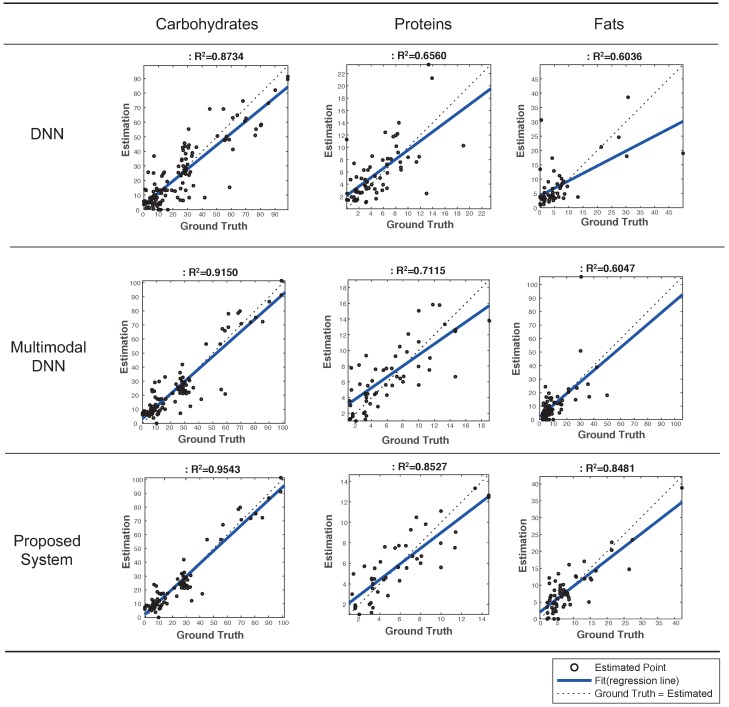
Evaluation of estimated vs. ground truth values as well as $R^{2}$ values in 3 systems to estimate food nutrients.

Figure 4 plots predicted vs. ground truth values for each of food nutrients, and presents $R^{2}$ values [33] that is a statistical measure of how good a regression model is; the higher the $R^{2}$ value, the better the model fits with the ground truth, and the value of 1.0 indicates a perfect fit. The system based on multimodal DNN had $R^{2}$ values of 0.9150, 0.7115, and 0.6047 for each of food nutrients, respectively. These values are 0.0011 ∼ 0.0555 higher than those of the simple DNN-based system. This result revealed that DNN and multimodal DNN had similar performance and that the degradation of accuracy in both systems was mainly caused by outliers or erroneous predictions.

By contrast, our proposed system successfully removed outliers, and hence, achieved $R^{2}$ values of 0.9543 (carbohydrates), 0.8527 (proteins), and 0.8481 (fats), and the average $R^{2}$ value was 0.885. Accordingly, our proposed system outperformed the multimodal DNN by 0.0393∼0.2434 in terms of $R^{2}$ values. This result demonstrated that it significantly improved the accuracy of estimating food nutrients by rejecting outliers. Clearly, the error avoidance scheme played a key role in achieving this promising result.

**Figure 5:**
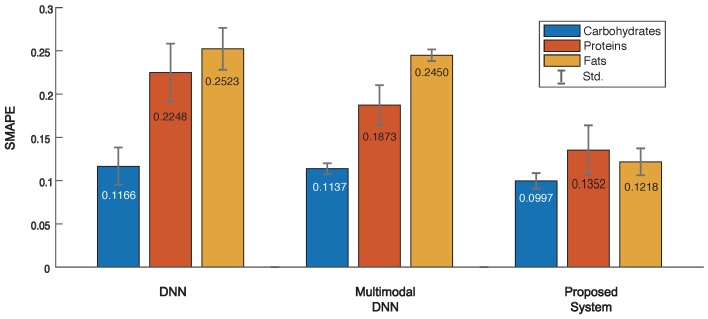
Evaluation of symmetric mean absolute percentage errors (SMAPE) in 3 systems to estimate food nutrients.

Figure 5 shows the results of average SMAPE evaluation of the best-five models on three systems. The DNN had an average SMAPE of 0.1166, 0.2248 and 0.2523 for CPF values, respectively, while the multimodal DNN received 0.1137, 0.1873, and 0.2450, respectively. This indicates the multimodal DNN performs slightly better than simple DNN in terms of SMAPE. In contrast, the proposed system achieved an average SMAPE of 0.0997, 0.1352, and 0.1218, respectively. This means the SMAPE of proposed system is 0.0169∼0.1305 and 0.0140∼0.1232 lower than the DNN and multimodal DNN, respectively. Obviously, this superior performance was due to the error avoidance scheme to reject outliers or erroneous predictions. As demonstrated, the proposed system is capable of accurately estimating food nutrients even from a new signal that was never seen before. However, if the input signal is the same as or similar to those in the database, vision- or RFID-based methods would be more effective thanks to the use of features (rather than raw hyperspectral signals) that can be easily distinguished. Hence one may want to employ a strategy to combine multiple methods to optimize the task of food analysis for the smart healthcare. Finally, we note that there exists a large number of recipes in reality, and hence, the proposed system may not perform well for a certain food. To lower the probability of misdetection, it is important to keep on training the system with more hyperspectral data.

## 4. Conclusions

By exploiting deep learning, this paper presented a novel system to precisely estimate the food nutrients (carbohydrates, proteins, and fats) from the measurements of hyperspectral signals. Two key building blocks of the proposed system are: (1) the multimodal architecture to extract common representation of features from the database of hyperspectral signals, and (2) the error avoidance scheme based on the autoencoder to automatically detect and reject estimated values with high errors. The results of our performance evaluation demonstrated that the proposed system accurately estimated CPF values as manifested by the average $R^{2}$ value of 0.885 and SMAPE value of 0.1189, while effectively suppressing estimation errors. The error avoidance scheme played a central role in achieving these results as it was able to filter out outliers or erroneous estimations, which could be very harmful to the patients or users. We envision that the accuracy of our proposed system keeps improving as we add more hyperspectral measurements for training.

## Figures and Tables

**Table 1:** List of 140 real foods in 5 categories.

Category	Num. Foods	Examples
Drink (juice)	37	Apple, tomato, orange, blueberry, lemon, grape, strawberry, sprite, coke, coffee, milk, ...
Sauce (spread)	10	Mustard, ketchup, mayonnaise, ...
Snack/candy/chocolate	19	Nacho, butter cookie, jelly, ...
Meat	8	Sausage, bacon, pork, ...
Miscellaneous	106	Grain (rice and bread), cheese, noodle, ...
